# A systematic review and meta-analysis of neuroticism and anxiety during the COVID-19 pandemic

**DOI:** 10.3389/fpsyt.2023.1281268

**Published:** 2024-01-05

**Authors:** Enkhtuvshin Regzedmaa, Mandukhai Ganbat, Munkhzul Sambuunyam, Solongo Tsogoo, Otgonbayar Radnaa, Nasantsengel Lkhagvasuren, Khishigsuren Zuunnast

**Affiliations:** ^1^Department of Mental Health, School of Medicine, Mongolian National University of Medical Sciences, Ulaanbaatar, Mongolia; ^2^Department of Epidemiology and Biostatistics, School of Public Health, Mongolian National University of Medical Sciences, Ulaanbaatar, Mongolia; ^3^School of Medicine, Mongolian National University of Medical Sciences, Ulaanbaatar, Mongolia; ^4^Department of Pediatrics, School of Medicine, Mongolian National University of Medical Sciences, Ulaanbaatar, Mongolia

**Keywords:** anxiety, COVID-19, neuroticism traits, systematic review, meta analysis

## Abstract

**Introduction:**

In response to the global impact of the COVID-19 pandemic, concerns about mental health, particularly anxiety levels, have become prominent. This study aims to explore the relationship between neuroticism, a personality trait associated with emotional instability, and anxiety during the COVID-19 outbreak.

**Methods:**

A comprehensive literature search was conducted using the Cochrane Library, HINARI, Google Scholar, and PUBMED, resulting in the identification of 26 relevant papers. The study protocol has been registered with PROSPERO under the number CRD42023452418. Thorough meta-analysis was performed using Comprehensive Meta-Analysis V4 software.

**Results:**

Meta-analysis revealed a significant positive relationship between anxiety and neuroticism, with 26 studies supporting this association (OR = 3.213, 95% CI 2.352 to 4.391). The findings underscore the importance of considering personality traits, particularly neuroticism, in understanding psychological responses to major global crises such as the COVID-19 epidemic.

**Discussion:**

The observed connection between neuroticism and heightened anxiety levels emphasizes the need for targeted interventions, especially for individuals with high levels of neuroticism. Further research into potential therapeutic approaches for mitigating anxiety consequences in the context of a significant global catastrophe is warranted.

**Systematic Review Registration:**

https://www.crd.york.ac.uk/prospero/#CRD42023452418.

## 1 Introduction

Numerous systematic reviews and meta-analyses have provided estimations of the prevalence of mental health outcomes during the COVID-19 pandemic (1–6). Several key concerns emerge from the available studies. Firstly, there is substantial increase in global anxiety prevalence post-COVID-19, affecting a considerable number of individuals, with a prevalence of 35.1% (7). This increased prevalence is consistent across low and middle-income countries to high-income countries (2). Secondly, systematic review and meta-analysis revealed that cognitive-behavioral therapy, training, and physical exercise interventions prove notably effective in addressing anxiety associated with the COVID-19 pandemic (8, 9). Lastly, the relationship between mindfulness as a trait and its associations with the Big Five personality traits and anxiety are explored (9–12). However, the synthesis of these relationships is not yet thoroughly examined in the context of the COVID-19 pandemic.

The Big-Five model is the most widely accepted model of personality. Five personality qualities are included: extraversion (to be sociable and active), agreeability (to be kind and trusting), conscientiousness (to be meticulous and dependable), emotional stability (to be at ease and peaceful), and openness (to be creative and curious) are the five personality traits (13). The most used tools on personal traits are Big Five Personality Traits and Eysenck Personality Questionnaire (14–32). Among the four types of personal traits, neuroticism and anxiety are significantly connected (14–44). People with high levels of neuroticism may be more prone to excessive worry and ruminating because they are more sensitive to threat indicators. It’s possible that neuroticism has a significant impact in shaping people’s anxiety responses given the pandemic’s inherent challenges and uncertainties (45). Several observational studies have investigated the relationship between neuroticism and anxiety during the COVID-19 pandemic (26, 29, 31, 38, 46, 47). In addition to that, while a comprehensive systematic review and meta-analysis explored the psychometric properties and psychological correlates of the COVID-19 anxiety syndrome scale on a broader scale (48), there is currently no systematic review specifically addressing the relationship between neuroticism and anxiety during COVID-19.

The current work seeks to fill this gap by undertaking a systematic review and meta-analysis of studies on the relationship between neuroticism and anxiety during the COVID-19 epidemic. This study tries to improve our understanding of the intricate interplay between personality traits and psychological reactions during times of crisis by combining the current empirical information. Furthermore, the findings of this study have significance for both clinical practice and public health initiatives, providing insights that can inform targeted strategies to support those who are especially sensitive to heightened anxiety during the pandemic.

## 2 Methods

### 2.1 Data sources and search strategy

To find relevant publications published between January 2020 and September 2023, a comprehensive literature search was done across key electronic databases such as MEDLINE (via PubMed), HINARI for access to research articles in developing nations and Google Scholar. Research protocol has been registered through protocol number CRD42023452418.

Search strategy: (“COVID-19” OR “coronavirus” OR “SARS-CoV-2”) AND (“anxiety” OR “stress” OR “psychological distress” OR “mental health”) AND (“personality traits” OR “neuroticism” OR “extraversion” OR “openness” OR “agreeableness” OR “conscientiousness”).

### 2.2 Inclusion and exclusion criteria

Studies considered in the systematic review satisfied following exposure-related inclusion requirements:

- Studies that measure exposure to neuroticism using standardized scales or questionnaires, such as the Big Five Inventory (BFI) or the NEO Five-Factor Inventory (NEO-FFI), are known as exposure studies. Known tools for measuring anxiety include the State-Trait Anxiety Inventory (STAI) and the Generalized Anxiety Disorder 7 (GAD-7) scale.
- Population: research on people who are affected by the COVID-19 epidemic, regardless of their age, gender, socioeconomic status, or location.

Studies that do not match the inclusion criteria or those that fall under the following categories was excluded:

- Studies that did not primarily examine the connection between neuroticism and anxiety during the COVID-19 epidemic were irrelevant in emphasis.
- Studies with insufficient data on measures of neuroticism and anxiety or those lacking the requisite statistical data are said to have incomplete data.
- Non-human studies: research involving animals or purely computer simulations or model systems.
- Articles, abstracts, conference proceedings, opinions, editorials, and non-systematic reviews that have not been peer-reviewed are considered non-peer-reviewed.
- Studies published in languages other than English due to a lack of resources for language translation.
- Research that was done before the COVID-19 pandemic.
- Duplicate data: to ensure data independence, studies having duplicate data from the same population and time was eliminated.
- Reviews, meta-analyses, and systematic reviews will be disqualified as non-original research. Only original research studies were considered.

#### 2.2.1 Extraction of data

Following authors (ER*, MG, KZ, MS, and ST) retrieved pertinent data from the selected papers in a methodical manner, including study characteristics, sample size, methodology, and major conclusion which is shown in Table 1.

**Table 1:** Studies included in the systematic review relationship between personality traits and anxiety during COVID-19.

Study author and year	Study design	Country	Population	Sample size	Personality traits examined	Personality traits measures	Anxiety measures	Main findings
Choi, 2023 (34)	Cross-sectional	United States	Students	132	Openness, conscientiousness, extroversion, agreeableness, and neuroticism	TIPI	STAI	Neuroticism is risk factor (*r* = 0.60, *p* < 0.001)
Fadime, 2022 (49)	Descriptive correlational study	Turkey	Students	360	Extroversion, agreeableness, conscientiousness, emotional stability, intelligence/imagination	IPIP	COVID-19 stress scale	Emotional stability is protective factor (*r* = −0.132*)
Ahmed, 2021 (50)	Cross-sectional online survey	Bangladeshi	>18 years old general population	531	Extraversion, agreeableness, conscientiousness, neuroticism, and openness	BFPI-10	FCV-19S	Inconsistent because, most of the studies have explored three latent profiles
Alexescu, 2022 (51)	Cross-sectional with pre-COVID-19 versus the COVID-19 period	Romania	Employees	138	Extraversion, neuroticism	EPI	None	Significant change before and after COVID-19
Árbol, 2021 (14)	Cross-sectional	Spain	Students	122	Neuroticism, extraversion, intolerance of uncertainty, problem solving focus, negative autofocus	EPQ-R	STAI	Neuroticism (0.524*), negative autofocus (0.551*) and intolerance of uncertainty (0.502*) are positive associated with anxiety
Belligntier, 2023 (33)	Cross-sectional	Germany	Adults	130	Extraversion, neuroticism	BFI-2	Coronavirus impact scale	Higher neuroticism was associated with greater perceived stress
Birkelund, 2023 (52)	Prenatal to postnatal period	Norway	Women	772 women-prenatally, 526-postnatally	Power, quality, stability, contacts	Human guide, a web-based psychological evaluation instrument	EPDS, GAD-7	The personality trait factors quality (*p* = 0.005) and contacts (*p* = 0.003) were significant predictors of anxiety
Bongelli, 2021 (53)	Cross-sectional	Italy	Frontline and non-frontline HCWs	682	Extraversion, agreeableness, conscientiousness, negative emotionality, open-mindedness	BFI-2-S	IPSS-10	No significant differences between frontline and non-frontline HCWs concerning personality traits, *F* (4, 2,720) = 1.664, *p* = 0.155
Cena, 2021 (54)	Web-based cross-sectional survey	Italy	Healthcare workers	235	Agreeableness, conscientiousness, emotional stability, extroversion, openness	BFI	IES-R	Higher emotional stability dimension of personality was associated with lower symptoms of pandemic related distress
Eroglu, 2023 (16)	Cross-sectional	Turkey	University students	720	Extraversion, agreeableness, conscientiousness, neuroticism, and openness to experience	BFI-10	Fear of COVID-19 scale	Only neuroticism is associated with fear of COVID-19 (*r* = 0.267)
Gashi, 2022 (15)	Cross-sectional	Republic of Kosovo	General population	200	Extraversion, neuroticism, openness to experiences, agreeableness and conscientiousness	BFI	ASR	Correlation between Big Five personality traits (compliance and neuroticism) and emotional problems (symptoms of anxiety)
Getzmann, 2021 (35)	Cross-sectional	Germany	General population	139	Neuroticism, extraversion, openness, agreeableness, and conscientiousness	NEO-FFI Personality Inventory	PSQ, TICS	Highly significant positive correlation of neuroticism and “worries,” *r* = 0.259; *p* < 0.001
Gruda, 2022 (17)	Cross-sectional	New York City	General population	1,336	Extraversion, neuroticism, openness to experiences, agreeableness and conscientiousness	BFI	STAI	Openness to experience (*r* = 0.01***), and neuroticism (*r* = 0.13***) are associated with anxiety
Kumar, 2022 (18)	Cross-sectional	India	General population	308	Extraversion, neuroticism, openness to experiences, agreeableness and conscientiousness	BFI	COVID-19 PAS	Neuroticism (*t* = 0.53), openness (*r* = 0.00), and agreeableness (*r* = 0.51) are associated with anxiety
Ikizer, 2022 (19)	Cross-sectional	Turkey	General population	99,217	Extraversion, neuroticism, openness to experiences, agreeableness and conscientiousness	BFI	Perceived Stress Scale	Neuroticism is positively associated with anxiety (*r* = 0.292***)
Joneghani, 2023 (20)	Cross-sectional	Iran	Women	130	(1) Extraversion, (2) adaptability, (3) conscientiousness, (4) neuroticism, and (5) openness	BFI Five Factor Scale	The Death Anxiety Scale	Neuroticism (*r* = 0.262***)
Kiziloğlu, 2023 (21)	Descriptive cross-sectional	Turkey	Nurses	325	Extroversion, neuroticism, and psychoticism.	EPQR-A	Fear of COVID-19 scale	Neuroticism (*r* = 0.240**)
Kluwe-Schiavon, 2022 (36)	Cross-sectional study	Portugal	Volunteers	722	Neuroticism, agreeableness, extraversion	NEO-PI-E	Depression, Anxiety and Stress Scale	Neuroticism (*r* = 0.12**)
Kong, 2021 (37)	Cross-sectional study	China	Medical staff	207	E (extraversion), N (neuroticism), P (psychoticism) and L (lie)	EPQ-RSC	SAAS, PANAS	Neuroticism (*r* = 0.330**)
Liu, 2021 (39)	Cross-sectional study	Canada	General population	1,055	Neuroticism, extraversion, conscientiousness	NEO-Five Factor Inventory	Self-reported question	Neuroticism (*r* = 0.48**)
Mazza, 2022 (22)	Cross-sectional study	Italy	General population	1,180	Openness to experience, agreeableness, extroversion, emotional stability/neuroticism, conscientiousness	BFI-10	SDQ	Neuroticism was positively related to the outcome (rGHQ = −0.318)
Metz, 2022 (23)	Cross-sectional study	United States	Dental residents	149	Extraversion, neuroticism, openness to experiences, agreeableness and conscientiousness	BFI	PTSD	Statistically significant difference neuroticism (OR = 2.9), conscientiousness (OR = 1.58), and PTSD
Mousavi, 2023 (24)	Cross-sectional study	Tehran	Hospitalized patients	160	Extraversion, neuroticism, openness to experiences, agreeableness and conscientiousness	BFI	PTSD	Neuroticism *r* = 0.53, openness to experience *r* = 0.18 are associated with PTSD
Nazari, 2023 (25)	Cross-sectional study	Indonesia	General population	728	Only neuroticism and extraversion	BFI-10	FCV-19S	Neuroticism *t* = 2.67
Norouzi, 2022 (41)	Cross-sectional study	Iran	General Population	225	Extraversion, neuroticism, openness to experiences, agreeableness and conscientiousness	BFI		Neuroticism *t* = 2.548
Olashore, 2021 (26)	Cross-sectional study	Africa	Patients	373	Neuroticism	44-item of BFI	The Anxiety Rating Scale	Neuroticism *t* = 0.516
Qian, 2020 (27)	Cross-sectional study	Japan	Yahoo users	2,000	Extraversion, neuroticism, openness to experiences, agreeableness and conscientiousness	Big-Five Scale	DASS	Neuroticism *r* = 0.141
Xu, 2023 (28)	Cross-sectional study	China	Intern students	181	Extroversion, agreeableness, conscientiousness, neuroticism and openness to experience	BFI-44	SAS	Neuroticism *r* = 0.429**
Agbaria, 2022 (55)	Cross-sectional study	Israeli-Palestinian	College students	625	Extraversion, emotional stability, openness to experiences, agreeableness and conscientiousness	BFPTSQ	Coping Style Questionnaire	Emotional stability is only positively associated with coping
Lassen, 2022 (29)	Cross-sectional study	Norway	Students	6,017	Extroversion, agreeableness, conscientiousness, neuroticism and openness to experience	BFI	PHQ-ADS	Neuroticism is positively associated with anxiety
Nikčević, 2021 (40)	Cross-sectional study	United States	General population	502	Extroversion, agreeableness, conscientiousness, neuroticism and openness to experience	BFI-10	PHQ	Neuroticism is positively associated with anxiety (*r* = 0.08)
Odachi, 2022 (30)	Cross-sectional study	Japan	General population	417	Extroversion, agreeableness, conscientiousness, neuroticism and openness to experience	BFS	FCV-19S	Neuroticism is positively associated with anxiety (*r* = 0.54)
Al-Omiri, 2021 (31)	Cross-sectional	Jordan	General population	1,319	Extroversion, agreeableness, conscientiousness, neuroticism and openness to experience	BFS	VAS	Neuroticism is significantly associated with anxiety
Taşci, 2022 (43)	Cross-sectional	China	Health and no-health community	451	Extroversion, agreeableness, conscientiousness, neuroticism and openness to experience	EPS_RCF	CAS	Neuroticism is positively associated with anxiety (*r* = 0.330)
Proto, 2021 (32)	Cross-sectional	United Kingdom	General population	5,583	Neuroticism (or emotional stability), extraversion, conscientiousness, agreeableness, and openness	BFS	GHQ-12	Neuroticism is significantly associated with anxiety

TIPI, The Ten Item Personality Inventory; STAI, The State Trait Anxiety Inventory; IPIP, 50-item International Personality Item Pool; BFPI-10, Ten item Big Five personality traits; FCV-19s, Fear of COVID-19 scale; EPI, Eysenck Personality Inventory; STAI, State-Trait Anxiety Inventory; EPQ-R, Eysenck Personality Questionnaire-Revised; EPDS, Edinburgh Postpartum Depression Scale; GAD-7, Generalized Anxiety Disorder-7; BFI-2-S, Big Five Inventory, Short Version; HCWs, Health Care Workers; IPSS10, Italian Perceived Stress Scale10; IES-R, Impact of Event Scale-Revised; ASR, The Adult Self-Report Questionnaire; PSQ, Perceived Stress Questionnaire; TICS, Trier Inventory for Chronic Stress; COVID-19 PAS, COVID-19 Pandemic Anxiety Scale; EPQR-A, Eysenck Personality Questionnaire-Revised Abbreviated Form; EPQ-RSC, Eysenck Personality Questionnaire Short Scale; SAAS, Social Appearance Anxiety Scale; PANAS, Positive and Negative Affect Scale; PTSD, Checklist for Diagnostic and Statistical Manual of Mental Disorders, 10th; DASS, Depression, Anxiety and Stress Scale; SAS, Self-Rating Anxiety Scale; BFPTSQ, Big Five Personality Trait Short Questionnaire; CAS, Coronavirus Anxiety Scale; EPS-RCF, Revised Eysenck Personality Survey-Shortened Form.

### 2.3 Data analysis

The Newcastle–Ottawa Scale was used to assess the quality and risk of bias of the included studies which was shown in Table 2.

**Table 2:** Quality assessment using the Newcastle–Ottawa Scale for assessing the quality and risk of bias of the included studies.

Study author	Selection^a^ (Max 4)	Comparability^b^ (Max 2)	Outcome^c^ (Max 3)	Total score (out of 9)
Árbol (14)	2	2	3	7
Kumar (18)	2	2	3	7
Lassen (29)	3	2	3	8
Nikčević (40)	3	2	3	8
Norouzi (41)	3	2	3	8
Odachi (30)	3	2	3	8
Xu (28)	2	2	3	7
Al-Omiri (31)	3	2	3	8
Nazari (25)	3	2	3	8
Proto (32)	3	2	3	8
Taşci (43)	3	2	3	8
Choi (34)	2	2	3	7
Eroglu (16)	2	2	3	7
Getzmann (35)	2	2	3	7
Gruda (17)	3	2	3	8
Ikizer (19)	4	2	3	9
Joneghani (20)	2	2	3	7
Kiziloğlu (21)	2	2	3	7
Kluwe-Schiavon (36)	2	2	3	7
Kong (37)	2	2	3	7
Liu (39)	3	2	3	8
Mazza (22)	3	2	3	8
Metz (23)	2	2	3	7
Mousavi (24)	2	2	3	7
Olashore (26)	2	2	3	7
Qian (27)	2	2	3	7

a Selection (representativeness of exposed/unexposed groups, ascertainment of exposure).

b Comparability (control for confounding factors).

c Outcome (assessment of outcome).

## 3 Results

### 3.1 Study selection

**Figure 1:**
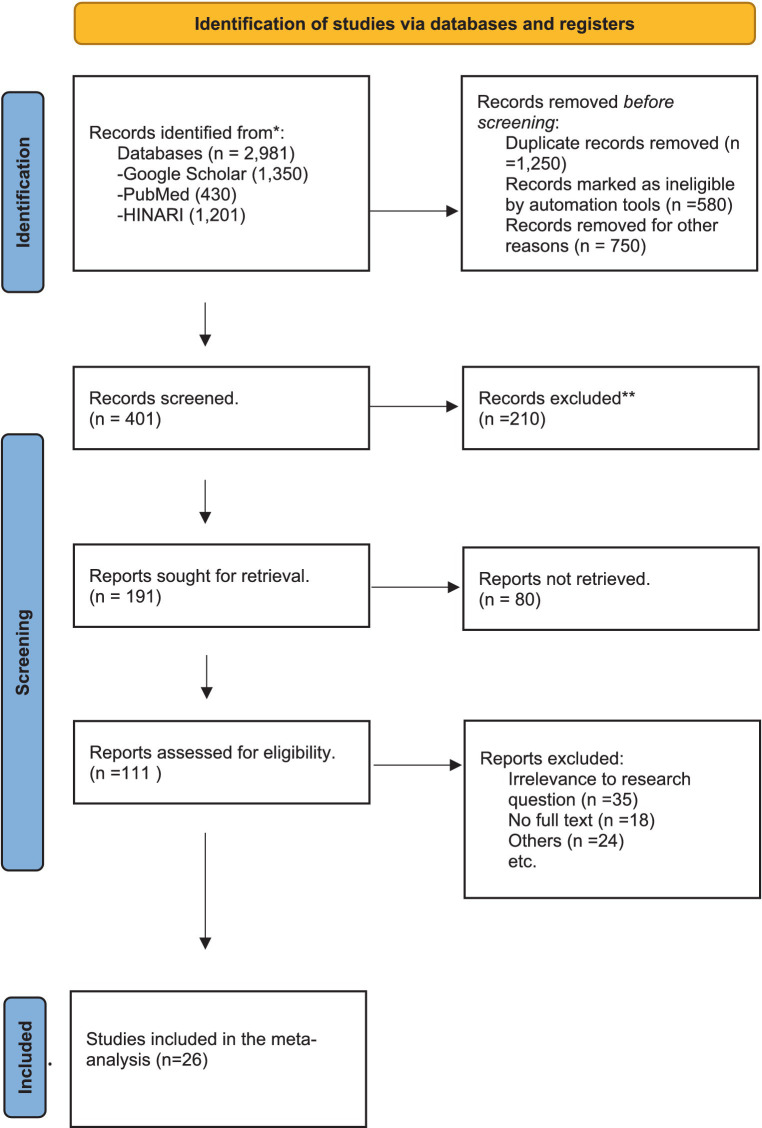
PRISMA 2020 flow diagram used for this systematic reviews which included searches of articles (56).

The procedure for a systematic review is shown in Figure 1. Five thousand nine hundred sixty-two documents were first found across several databases. Two thousand nine hundred eighty-one of them came from PubMed and Hinari, and 1,350 from Google Scholar when appropriate keywords were used. One thousand two hundred fifty duplicate records were eliminated, and then automation was used to mark 580 records as invalid. A further 750 records were excluded for factors other than duplication or eligibility. Four hundred one records underwent screening, with titles and abstracts being examined for appropriateness.

Two hundred ten of these data were left out because they did not suit the study’s parameters or subject. One hundred ninety-one records from the screened records were chosen for further analysis. Eighty retrievals were made, but none were successful, probably owing to access restrictions. One hundred eleven of the collected papers underwent an extensive eligibility review. Of these, 24 were removed for predetermined reasons, such as methodological difficulties, and 24 were deemed irrelevant, 18 did not have full-text access.

Ultimately, 26 eligible papers were picked for the meta-analysis following thorough examination. The Newcastle–Ottawa risk of bias evaluation determined that these studies satisfied its requirements. This procedure makes sure that the final choice is trustworthy and pertinent to the goal of the investigation.

### 3.2 Characteristics of included studies

Table 1 summarizes the characteristics of the included studies. The studies were conducted between 2020–2023 and encompassed 36 geographical regions such as United States, Turkey, Bangladesh, Romania, Spain, Germany, Norway, Italy, Republic of Kosovo, New York City (United States), India, Isfahan (Iran), Portugal, China, Canada, Tehran (Iran), Indonesia, Iran, Africa, Japan, Israeli-Palestinian, Norway, United States, Jordan, United Kingdom, and China with sample sizes ranging from min 130 to 99,217 max sample size. Most studies employed a cross-sectional study design, and the populations under investigation included list populations, along with students, people over the age of 18 in the general population, employees, adults, women, frontline and non-frontline healthcare workers, nurses, volunteers, medical staff, dental residents, hospitalized patients, Yahoo users, intern students, and college students. The outcomes/exposures evaluated in these studies varied.

### 3.3 Risk of bias assessment

**Figure 2:**
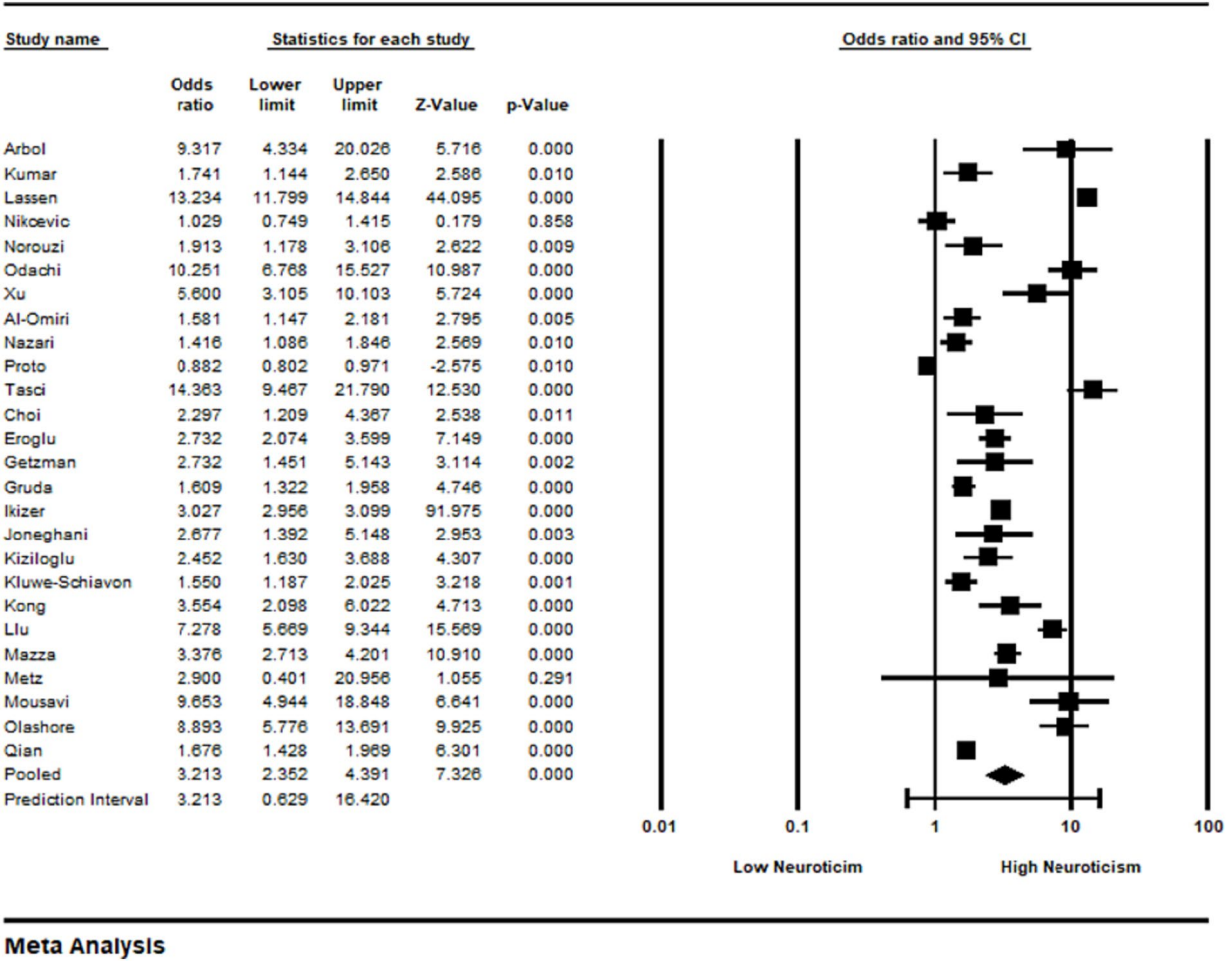
Systematic review of the included studies.

The risk of bias assessment results for individual studies are presented in Figure 2. Studies were evaluated for potential biases using the Newcastle–Ottawa Scale is a frequently employed instrument for evaluating the caliber of non-randomized research in meta-analyses by study authors OR and NL. It assesses three key areas: exposure ascertainment, group comparability, and study group selection. Based on criteria within these domains, the scale provides stars to each study, allowing users to assess the inclusion of studies’ methodological quality and bias risk. Table 2 gives a thorough breakdown of the Newcastle–Ottawa Scale quality evaluation performed for the included research, evaluating both the quality and risk of bias. The ratings are based on how well the selection (maximum score of 4) and comparability (maximum score of 2) and outcome assessment (maximum score of 3) criteria were evaluated. A number of notable studies, including Joneghani and Sajjaian (20), Árbol et al. (14), Kumar and Tankha (18), and Xu et al. (28), got a total score of 7, while others, such Ikizer et al. (10) received the highest total score of 9, indicating solid quality and minimal danger of bias. The three main criteria of the scale—selection, comparability, and outcome—are applied to assess the methodological merits and limitations of the studies, resulting in a more thorough comprehension of their validity and reliability in examining the relationship between neuroticism and anxiety during the COVID-19 pandemic.

### 3.4 Quantitative synthesis (meta-analysis)

A forest plot of the pooled effect estimates for anxiety is shown in Figure 2. The meta-analysis of 26 studies investigating anxiety revealed an odds ratio, 95% CI. Comprehensive Meta-Analysis Software has been used to analyze meta-analysis. Our meta-analysis used a random-effects model. The random-effects model was used because it assumes that variations in population, methodology, or other factors may cause the true impact magnitude to differ between studies. This method takes possible heterogeneity into consideration and is more conservative.

To evaluate heterogeneity among the included studies, we computed the *I*^2^ statistic. Significant heterogeneity was observed in the results [*p* < (0.0001), and *I*^2^ = (48%)].

The heterogeneity across studies was assessed using the *I*^2^ statistic and was found to be heterogeneity value *I*^2^ = 48% indicating moderate heterogeneity. Figure 2 shows a forest plot with the combined effect size for the relationship between neuroticism and anxiety. An intensely positive association between anxiety and neuroticism was found by meta-analysis, which was supported by 26 number of research (OR = 3.213, 95% CI 2.352 to 4.391). The findings of the systematic review as shown in Figure 2 provide a thorough summary of the correlation coefficients and statistical parameters obtained from several studies evaluating the connection between personality characteristics and anxiety during the COVID-19 epidemic. Among other noteworthy results, Árbol’s et al. (14) study showed a substantial positive association (0.524) between neuroticism and anxiety among Spanish students. In a similar vein, Lassen’s research (29) on a sizable student sample shows a substantial correlation coefficient of 0.58 between particular personality qualities and anxiety. The study also emphasizes the importance of correlation coefficients in research like Xu’s et al. (28), where neuroticism and anxiety in intern students are reported to have a substantial association of 0.429. Similar findings are shown in Ikizer’s et al. (19) study, which shows that neuroticism and anxiety have a moderate connection (0.292) in a sizable general sample. Notably, odds ratios are introduced as pertinent indicators in Metz’s et al. (23) research. Together, the results of this systematic review help us gain a more nuanced understanding of the complex relationship that exists between personality traits and anxiety in the context of the difficulties brought on by the COVID-19 epidemic.

## 4 Discussion

Our meta-analysis has found that there is a positive relationship between neuroticism and anxiety during the COVID-19 pandemic. This result is consistent with earlier theoretical models that contend there is a close connection between these two notions (14, 18, 20, 24, 25, 29–32, 34, 35, 40, 41, 44, 45). An extensive systematic review and meta-analysis, involving the examination of 17,789 individuals, demonstrated that anxiety was positively correlated with neuroticism, but inversely correlated with extraversion. This study is also limited by its study design and lacks information about the pre- and post-COVID-19 pandemic period (48). However, 134 cohort including systematic review and meta-analysis showed that no changes were found for general mental health (57). The highest anxiety prevalence during COVID-19 was found among health care workers (6). Our systematic review did not focus on subgroup analysis. However, women displayed higher scores on anxiety during COVID-19 (47). Initially, our study wanted to subgroup analysis by lower and higher resource setting, but this study did not find any significant differences when we deal with anxiety during COVID-19 (26). Neuroticism is associated with emotional risk during the COVID-19 pandemic. Those high in neuroticism tend to pay more attention to COVID-19 information and worry extensively about its consequences (crisis preoccupation) (38).

Personality traits were found to be correlated with the effects of COVID-19. The significance of the relationship between personality traits and COVID-19-related changes is illustrated by these results (31). Cross-sectional online survey, utilizing the German version of the COVID Stress Scales (CSS) and standard psychological questionnaires highlight neuroticism as a risk factor and extraversion as a protective factor influencing pandemic-related stress responses (58).

Although our research provides valuable insights into the correlation between neuroticism and anxiety amidst the COVID-19 pandemic, it is important to acknowledge its inherent limitations. When interpreting the results, it is crucial to consider these limitations, which also indicate potential areas for future research to improve:

- Study design and temporal data deficiency: the experimental nature of the study restricts our capacity to establish causation, as it merely provides associations rather than causal connections.
- Temporal limitations: the lack of data prior to and following the pandemic hinders the development of a comprehensive comprehension of the relationship between neuroticism and anxiety over an extended period. To overcome this constraint, longitudinal designs may be implemented.
- Disparity among research studies: the presence of substantial heterogeneity (*I*^2^ = 48%) suggests that there is considerable variation among the studies. Potential sources of variation include disparities in populations, approaches, or uncontrolled variables, all of which may influence the dependability of the aggregate effect estimates.
- Analysis of subgroups and resource configurations: the research did not conduct an in-depth examination of subgroup dynamics, such as variations in resources and demographics. The consideration of subgroup-specific subtleties may yield supplementary perspectives.
- Gender bias: although the research identified elevated anxiety levels among women, the exclusive attention to gender-specific trends might restrict the applicability of the results to more extensive demographic cohorts.
- Definitions of resource setting variations: the preliminary objective of examining variations in anxiety levels in relation to resource settings produced inconclusive findings. Subsequent investigations ought to thoroughly examine this facet.
- Bias in publications: publication bias may arise due to the possibility that studies with significant findings will be selectively reported, which could have an impact on the overall effect estimate.
- Regional and cultural particularities: the limited presence of cultural diversity in the literature under analysis may impose restrictions on the generalizability of the study. Further investigation is warranted to examine the extent to which the association between neuroticism and anxiety differs across cultures in the context of pandemics.
- The presence of variability in the psychometric instruments utilized across studies could potentially introduce inconsistencies in measurements, which could have an adverse impact on the precision of the aggregated effect estimates.
- Insights into limited interventions: the research predominantly investigates correlations, thereby offering restricted perspectives on intervention strategies. Subsequent investigations ought to address this knowledge deficit by examining efficacious approaches to alleviate anxiety associated with neuroticism.
- External considerations regarding validity: the findings of this study may be limited in their applicability to different crisis contexts or stressors due to its narrow focus on the COVID-19 pandemic.
- Exclusive emphasis on neuroticism: the research primarily centers on neuroticism, disregarding the potential ramifications of multiple factors influencing anxiety. Further investigation is warranted to examine an even broader spectrum of contributing factors.

Further research should utilize longitudinal designs to examine the temporal dimensions of the relationships between neuroticism and anxiety over an extended period. This would enable a more comprehensive understanding of the dynamics underlying these constructs. By adopting this methodology, valuable insights could be gained regarding the long-term course of psychological reactions following the acute phase of the pandemic. Comprehensive subgroup analyses encompassing a wide range of demographic variables, including but not limited to age, socioeconomic standing, and cultural distinctions, may reveal intricate patterns within the correlation between neuroticism and anxiety. Gaining insight into the way these variables interact with individual personality traits can inform community-specific interventions. An examination of protective factors, in addition to extraversion, may contribute to the advancement of knowledge regarding resilience in the face of adversity. The exploration of factors that alleviate the effects of neuroticism on anxiety may provide valuable insights for the development of interventions and support systems that aim to improve psychological health.

Conducting comparative studies across various global regions may shed light on cultural differences in the correlation between neuroticism and anxiety in the context of pandemics. Conducting cross-cultural research has the potential to unveil unique coping strategies and reactions, thereby enhancing our overall comprehension of whether these associations are universal or culturally specific.

The significance of incorporating neuroticism into anxiety intervention design, especially in times of crises such as the COVID-19 pandemic, is highlighted by our findings. Personalized therapeutic strategies that target the distinct obstacles encountered by individuals with elevated levels of neuroticism might prove to be more efficacious in alleviating symptoms associated with anxiety. By incorporating the findings of this study into their messaging and public health campaigns, communication strategies that effectively connect with individuals who exhibit high levels of neuroticism can be developed. Developing communications that offer reassurance, trustworthy information, and coping mechanisms could prove to be especially advantageous for this specific demographic. The integration of our findings into clinical practice can be achieved by mental health professionals through the integration of neuroticism assessments during the evaluation of anxiety. By incorporating personalized treatment plans that recognize the impact of neuroticism on anxiety, therapeutic interventions can be rendered more efficacious. Community support programs that seek to enhance mental well-being should contemplate the customization of assistance services to cater to the needs of individuals who exhibit elevated levels of neuroticism. To address the unique requirements of this demographic, these programs may encompass focused counseling sessions, seminars on stress management, and community-building exercises.

In brief, forthcoming research ought to further investigate the intricacies of the correlation between neuroticism and anxiety by utilizing a variety of methodologies and considering a broad spectrum of influential factors. The practical ramifications underscore the necessity for focused interventions and approaches that can be applied in public health, clinical, and community environments to assist people experiencing crises who exhibit differing degrees of neuroticism.

## Data availability statement

The original contributions presented in the study are included in the article/supplementary material, further inquiries can be directed to the corresponding author.

## Author contributions

KZ: Writing – original draft, Writing – review & editing. ER: Formal analysis, Writing – review & editing, Conceptualization, Investigation, Methodology, Writing – original draft. MG: Software, Formal analysis, Validation, Writing – review & editing. MS: Data curation, Software, Investigation, Writing – original draft. ST: Data curation, Investigation, Software, Writing – original draft. OR: Writing – review & editing. NL: Writing – review & editing.
