# Erratum: A systematic review and meta-analysis of neuroticism and anxiety during the COVID-19 pandemic

**DOI:** 10.3389/fpsyt.2024.1382413

**Published:** 2024-02-14

**Authors:** Frontiers Production Office

**Affiliations:** Frontiers Media SA, Lausanne, Switzerland

**Keywords:** anxiety, COVID-19, neuroticism traits, systematic review, meta analysis

Due to a production error, the order of authors was incorrect. The correct order is as follows:

Enkhtuvshin Regzedmaa, Mandukhai Ganbat, Munkhzul Sambuunyam, Solongo Tsogoo, Otgonbayar Radnaa, Nasantsengel Lkhagvasuren, and Khishigsuren Zuunnast.

The publisher apologizes for this mistake. The original version of this article has been updated.

